# Supplementary figures and images for: Real-time infectious disease endurance indicator system for scientific decisions using machine learning and rapid data processing
Source: PeerJ Comput Sci. 2024 Jul 30;10:e2062. doi: 10.7717/peerj-cs.2062 (PMC11323025; doi:10.7717/peerj-cs.2062)

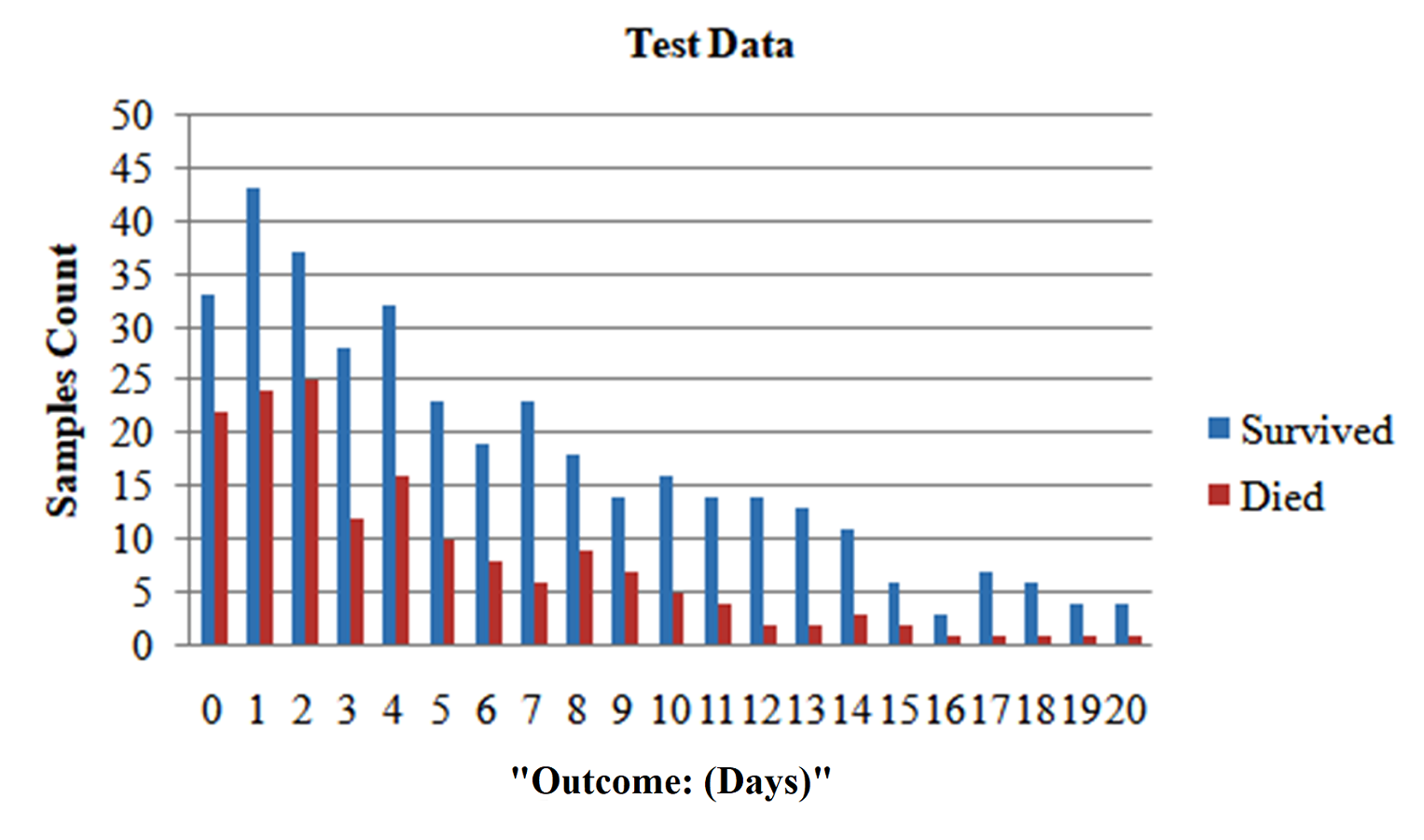

Supplement: Supplemental Information 7 [file peerj-cs-10-2062-s007.zip › Figure 2.png]

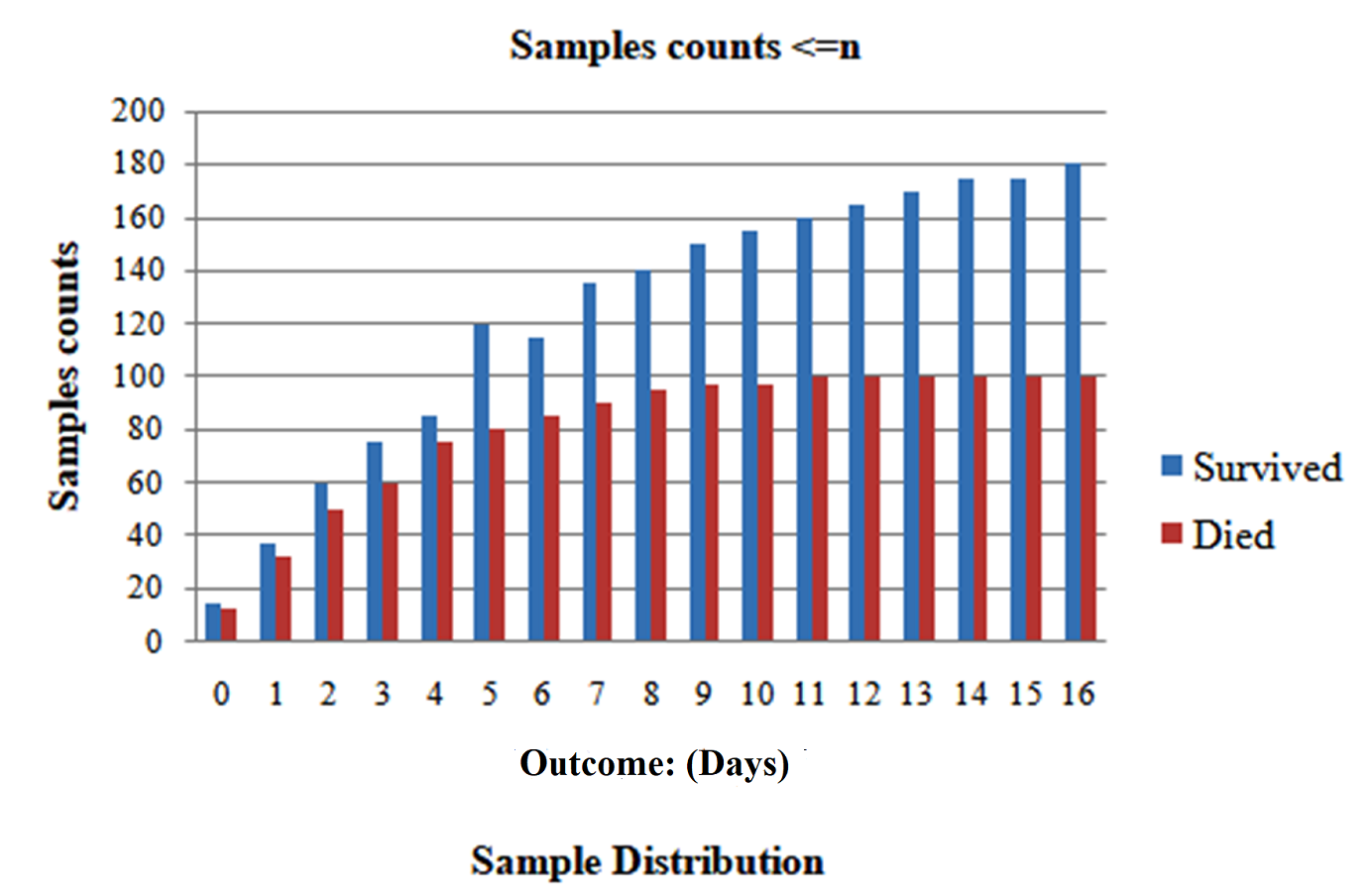

Supplement: Supplemental Information 7 [file peerj-cs-10-2062-s007.zip › Figure 7.png]

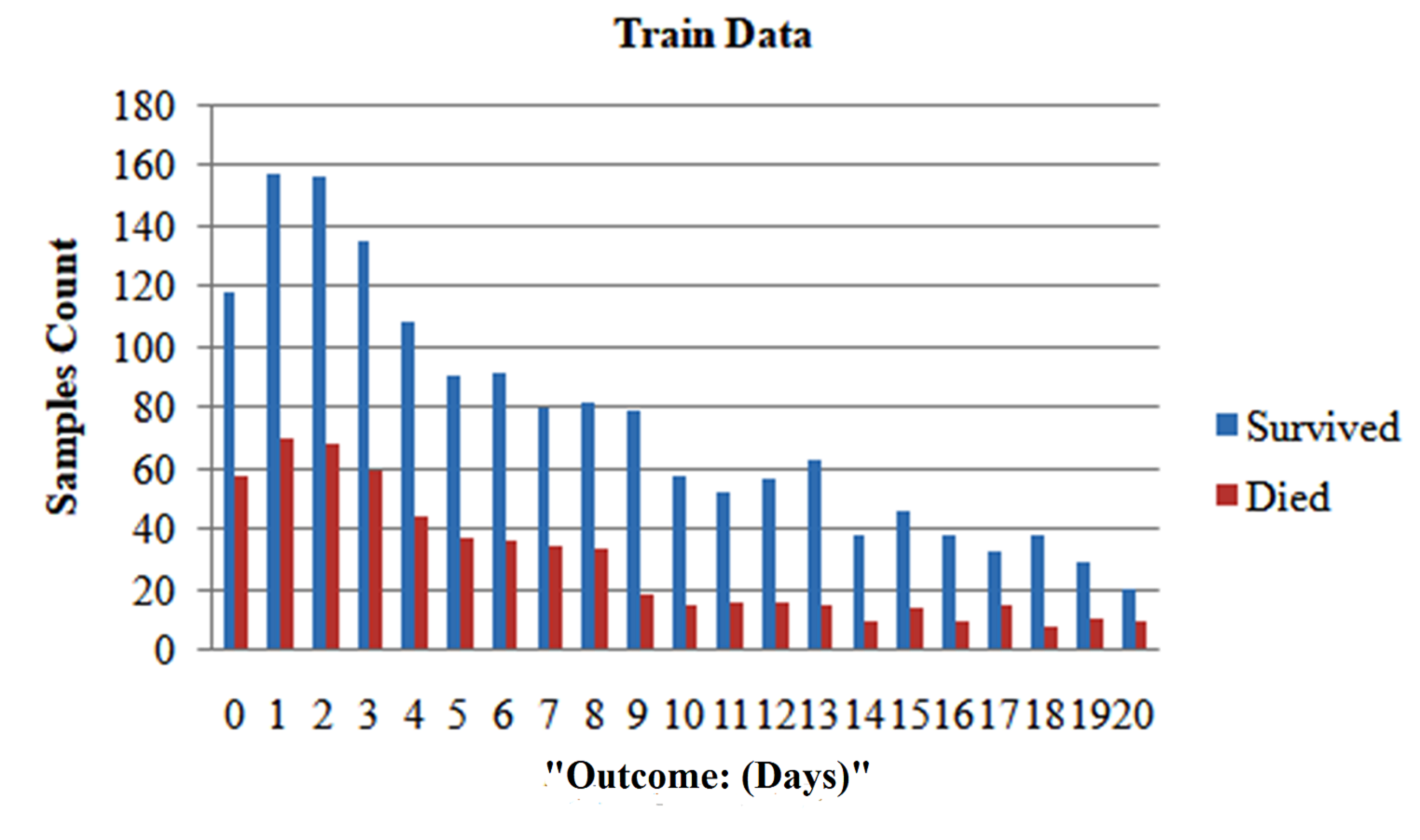

Supplement: Supplemental Information 7 [file peerj-cs-10-2062-s007.zip › Figure 1.png]

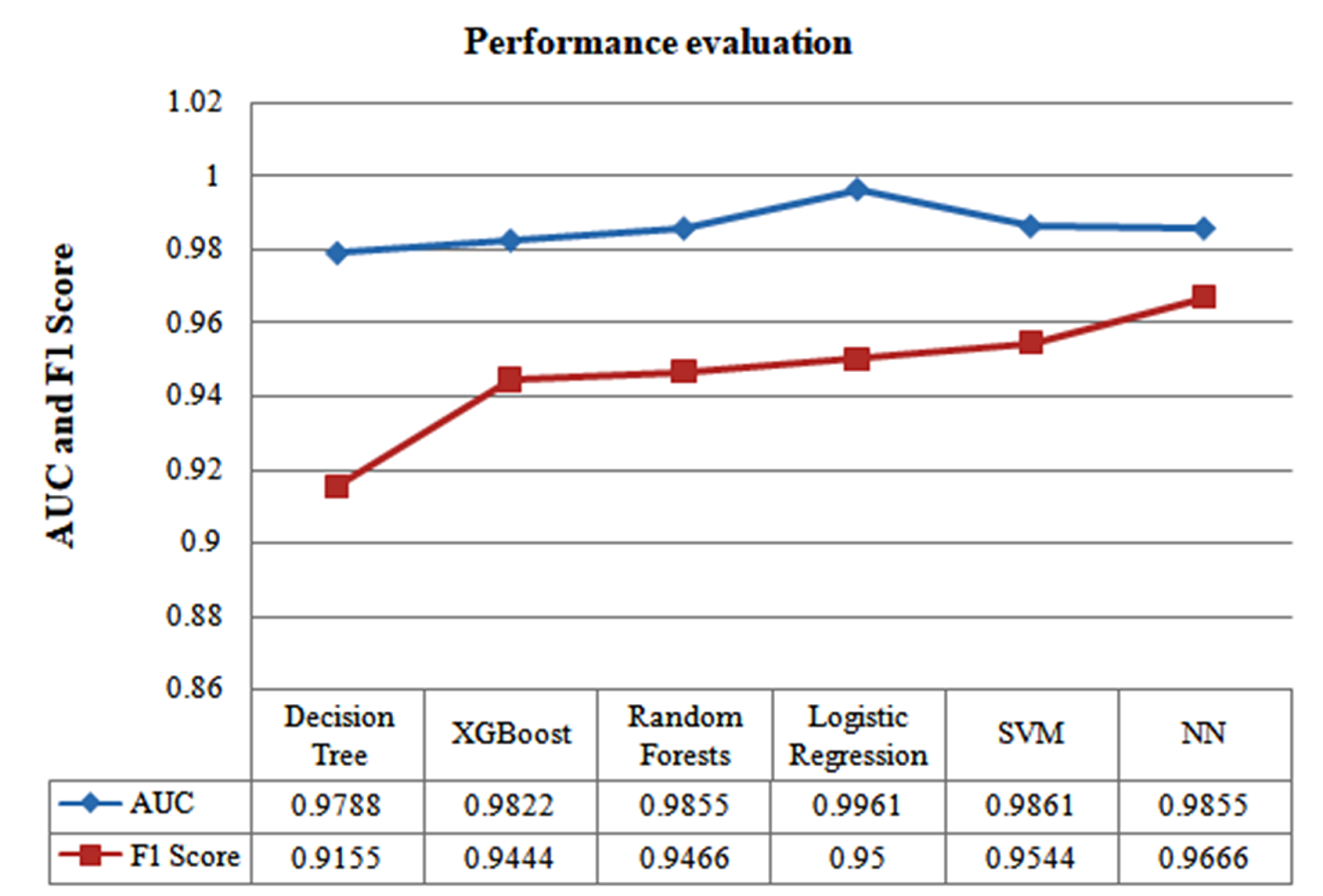

Supplement: Supplemental Information 7 [file peerj-cs-10-2062-s007.zip › Figure 6.png]

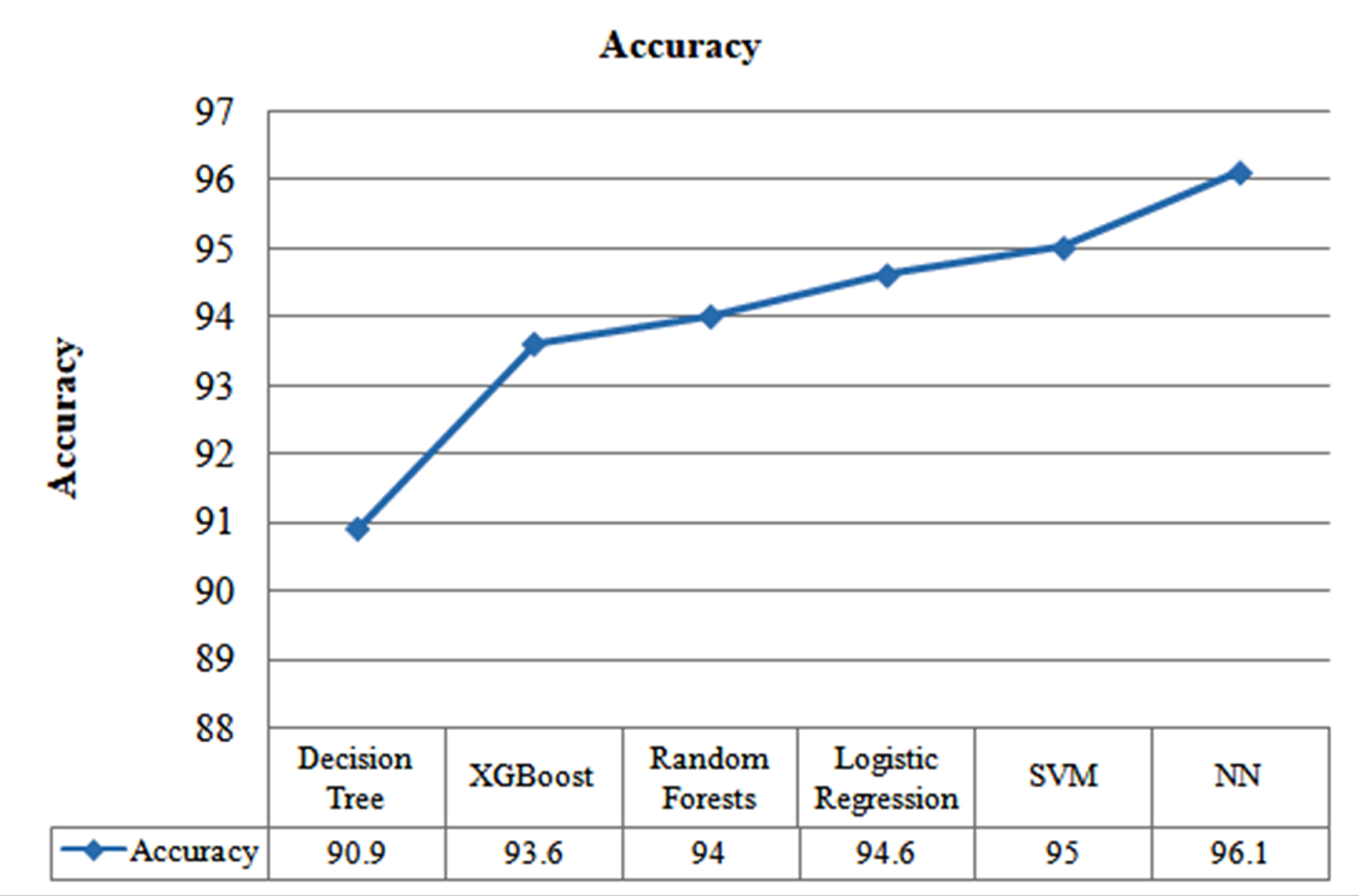

Supplement: Supplemental Information 7 [file peerj-cs-10-2062-s007.zip › Figure 5.png]

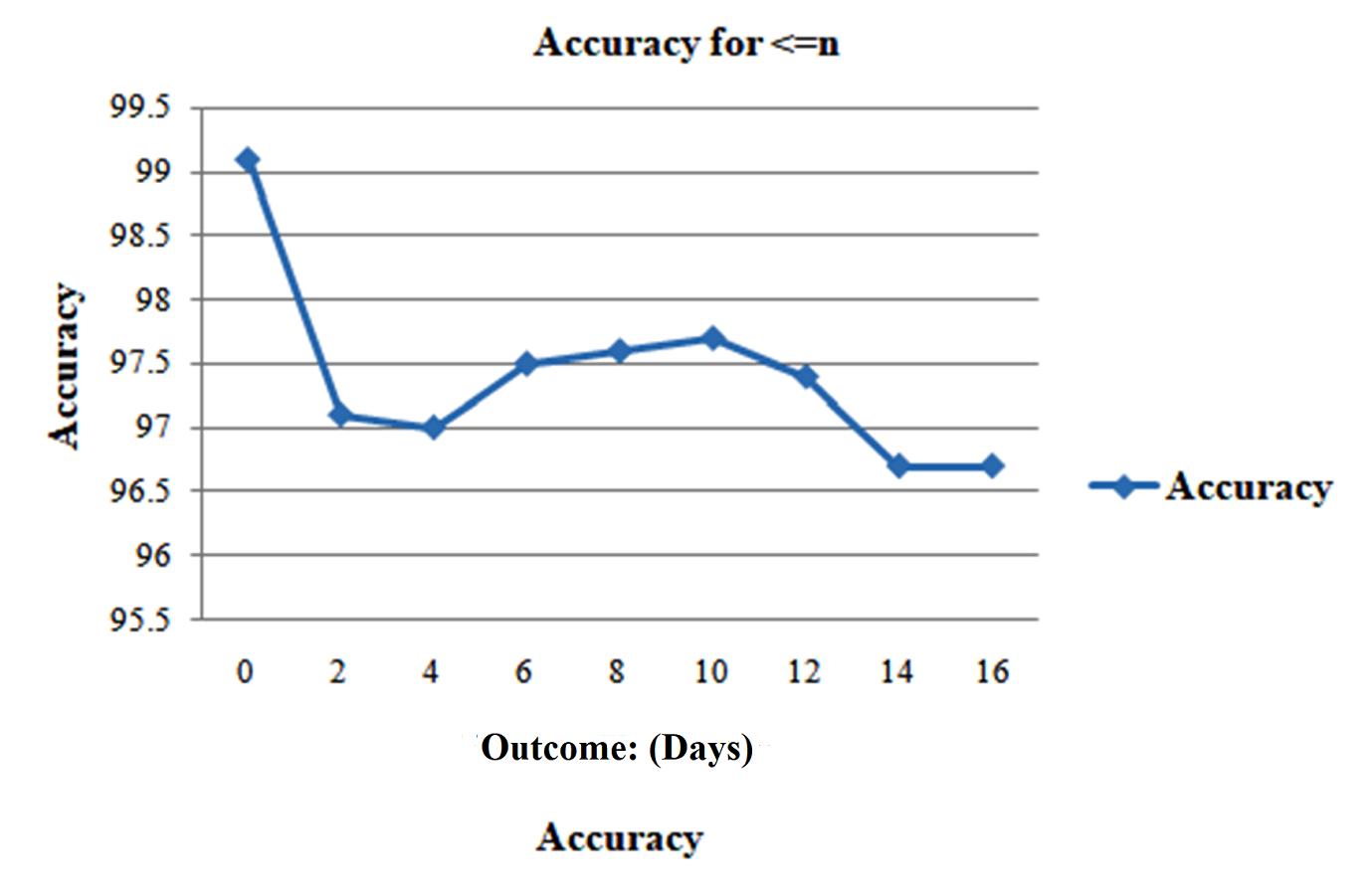

Supplement: Supplemental Information 7 [file peerj-cs-10-2062-s007.zip › Figure 8.png]

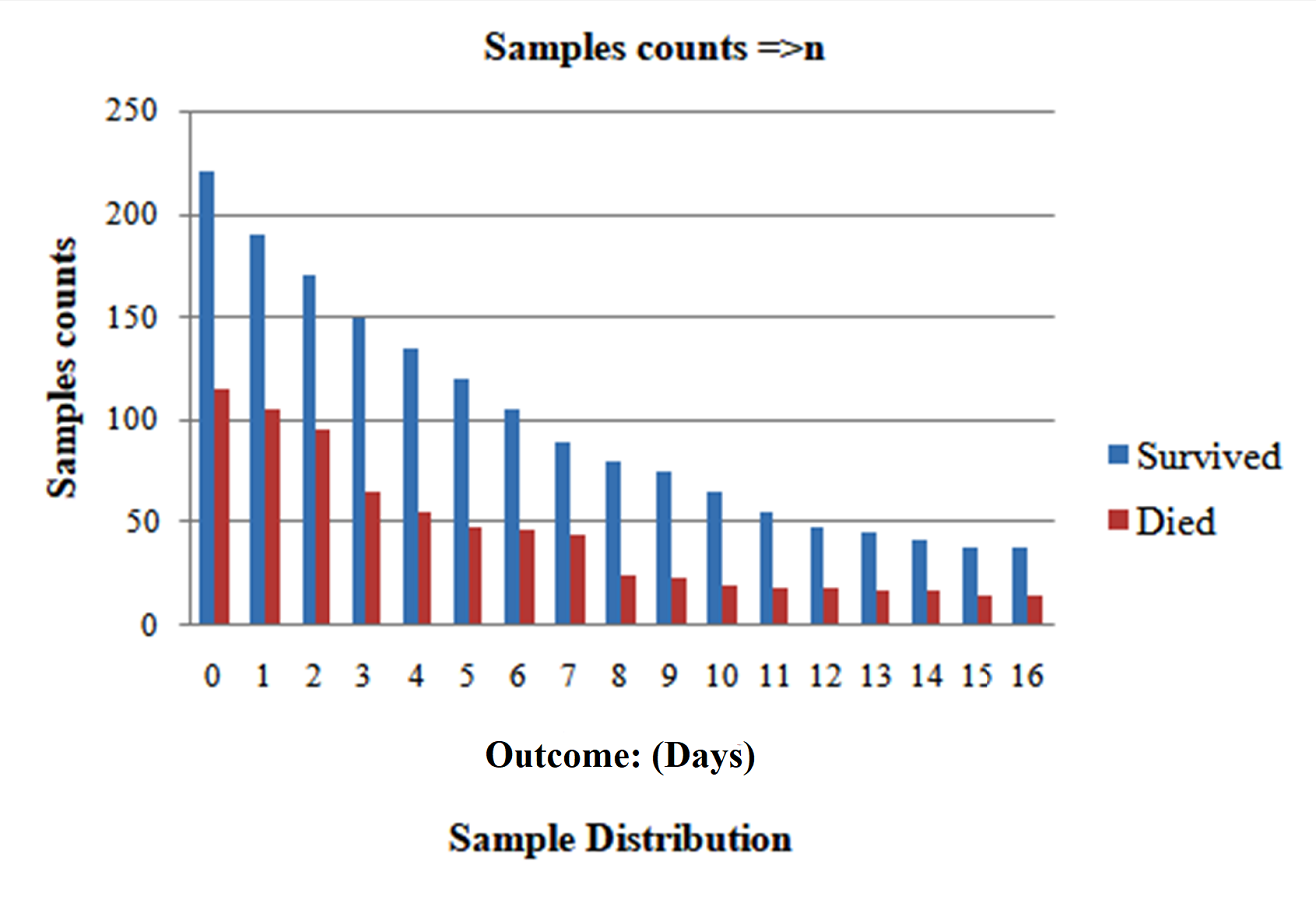

Supplement: Supplemental Information 7 [file peerj-cs-10-2062-s007.zip › Figure 10.png]

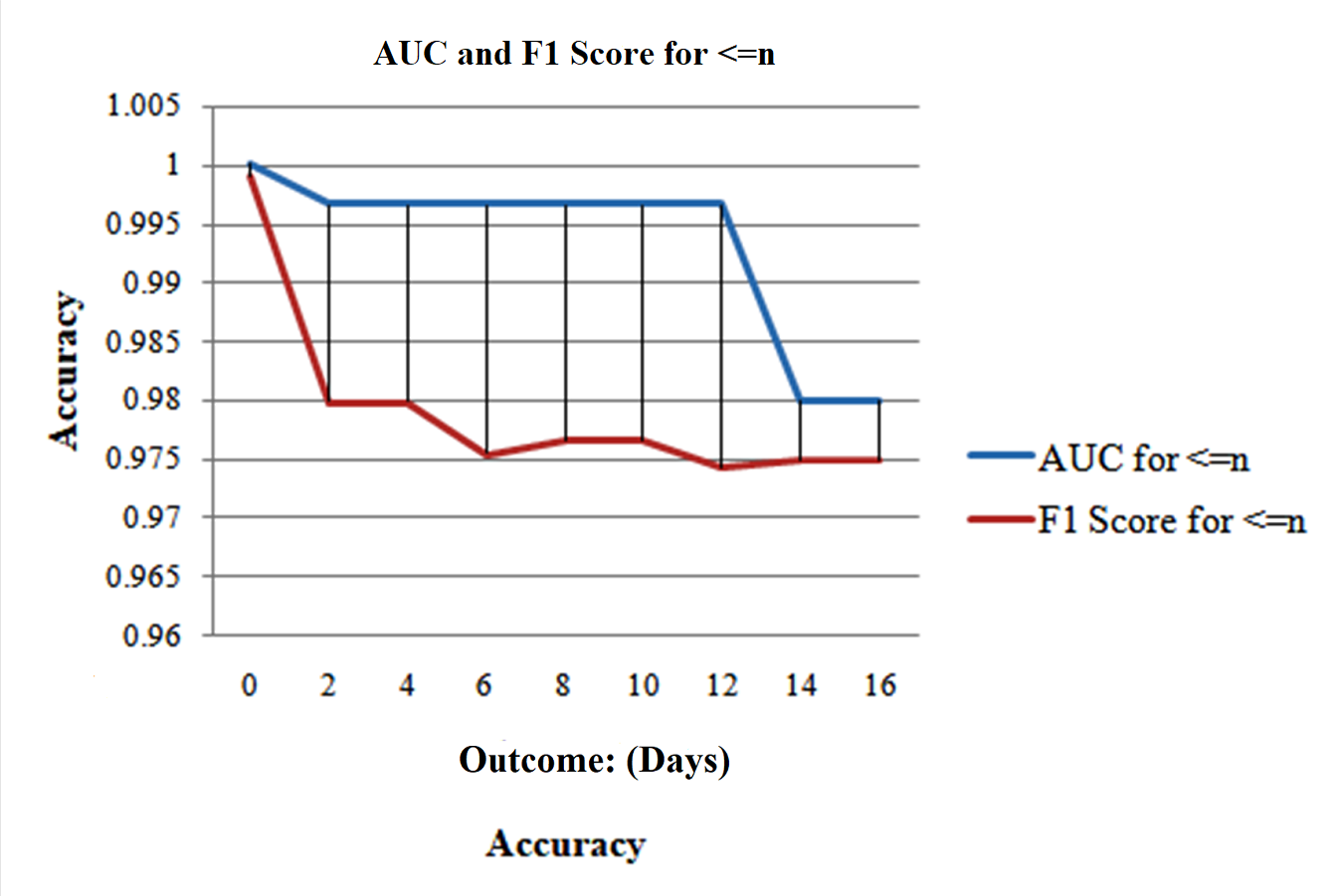

Supplement: Supplemental Information 7 [file peerj-cs-10-2062-s007.zip › Figure 9.png]

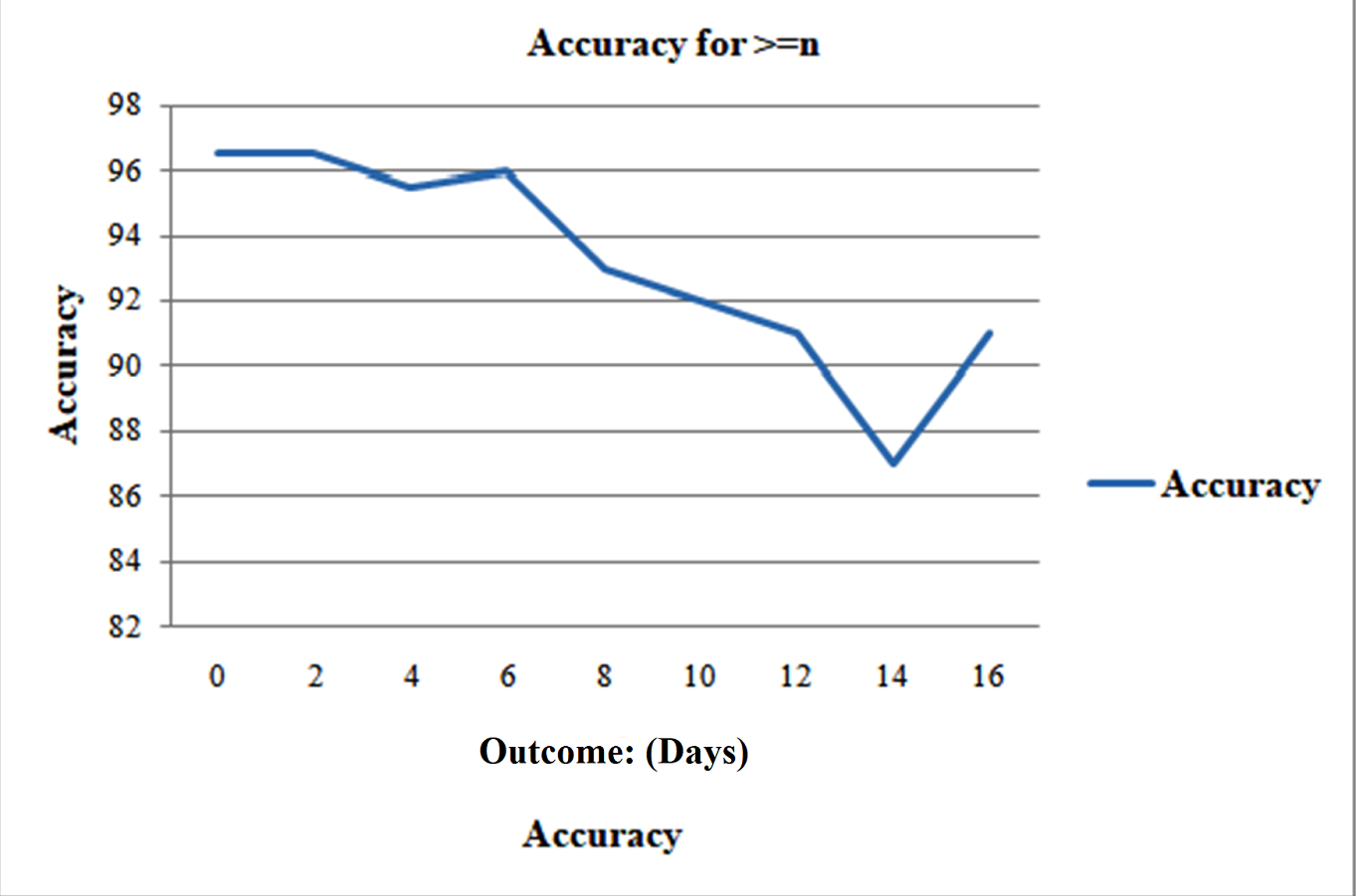

Supplement: Supplemental Information 7 [file peerj-cs-10-2062-s007.zip › Figure 11.png]

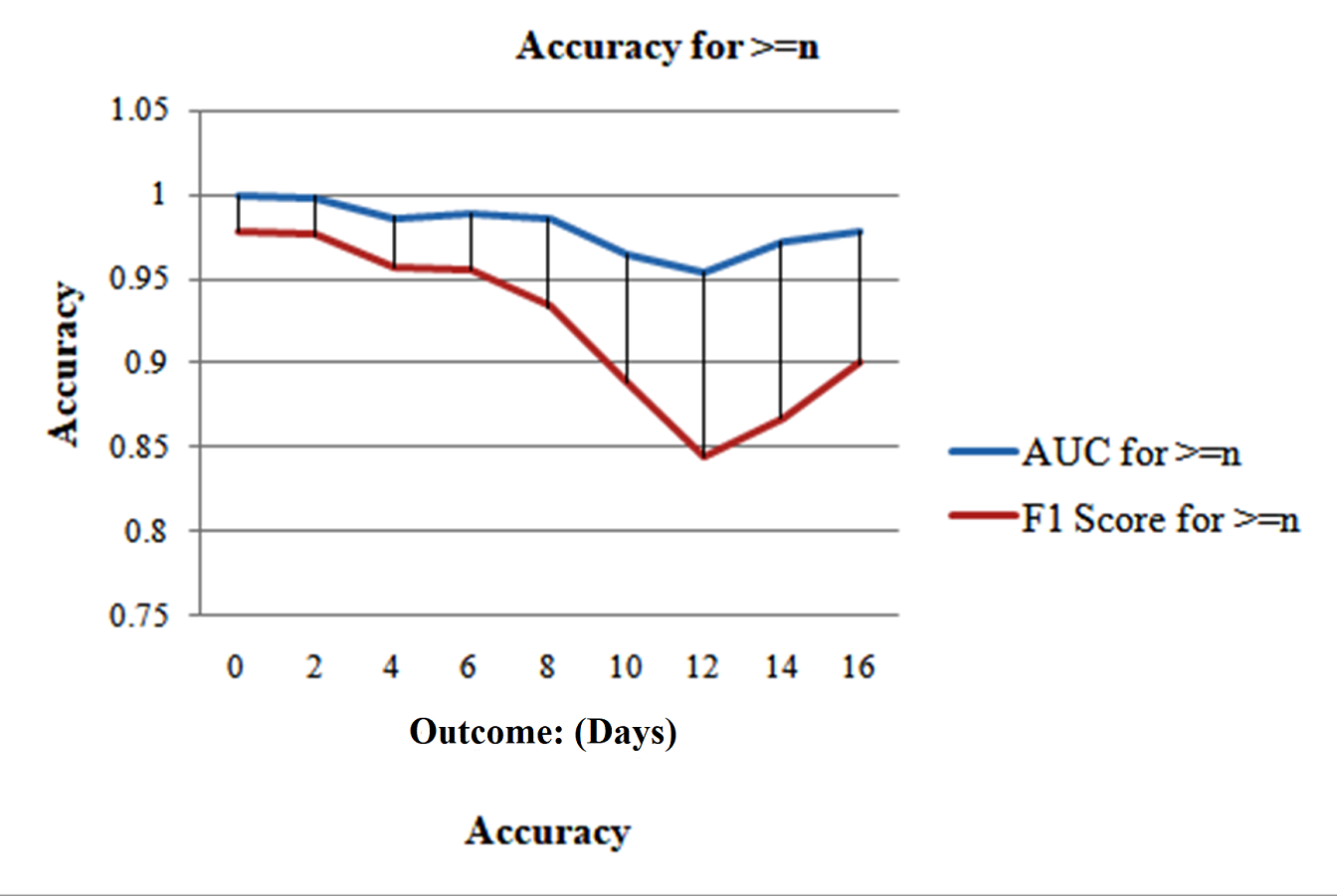

Supplement: Supplemental Information 7 [file peerj-cs-10-2062-s007.zip › Figure 12.png]

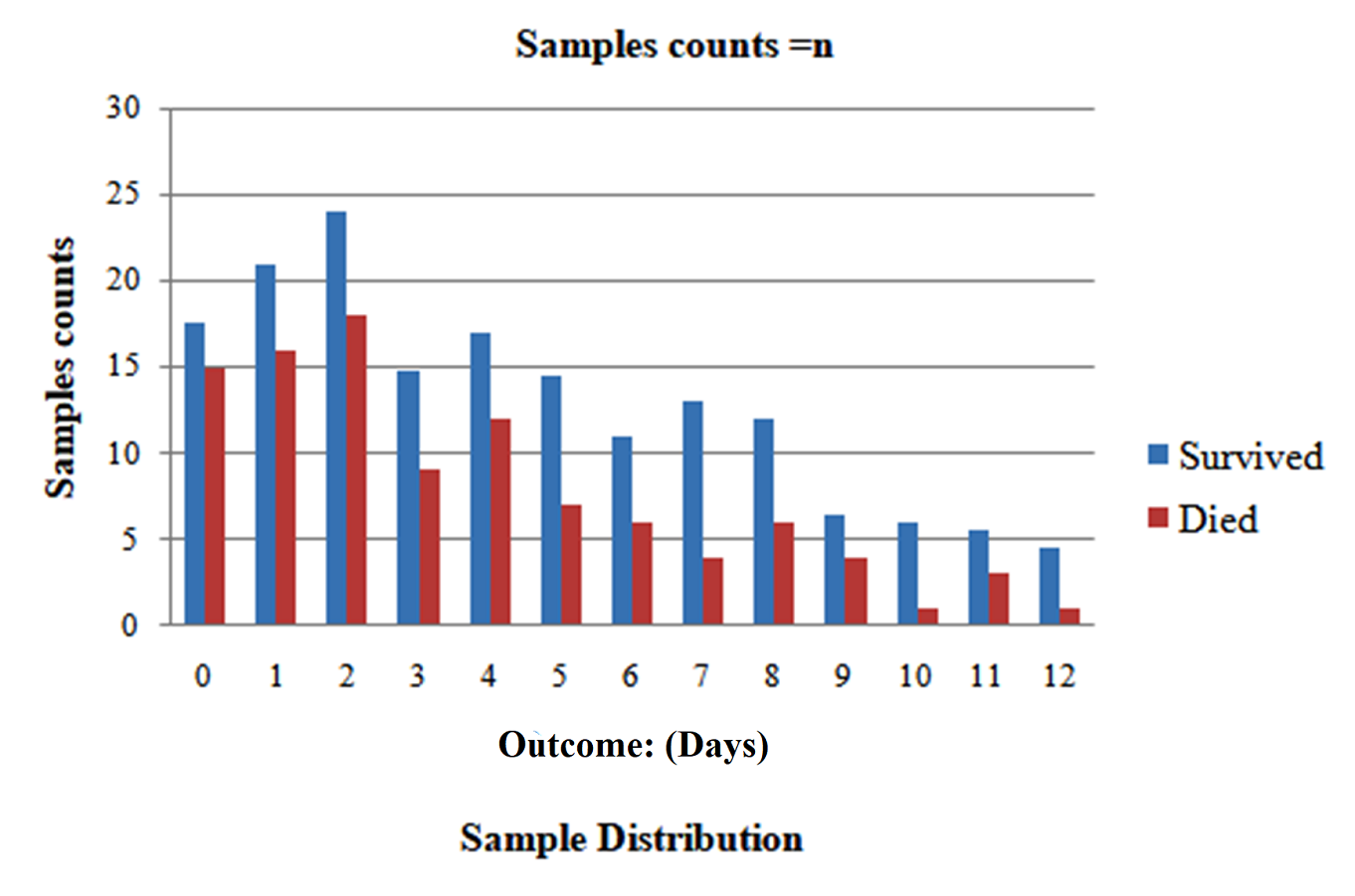

Supplement: Supplemental Information 7 [file peerj-cs-10-2062-s007.zip › Figure 13.png]

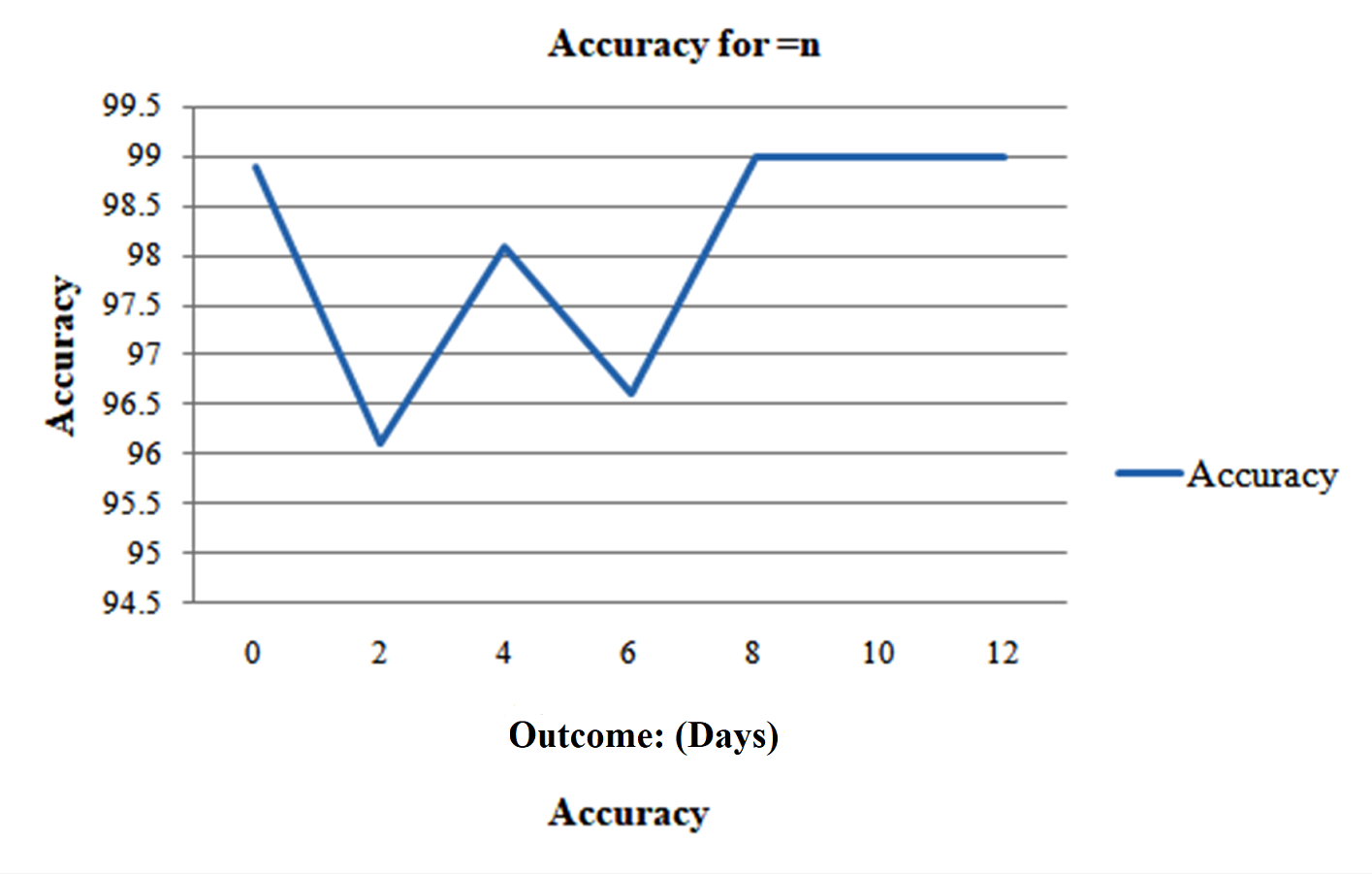

Supplement: Supplemental Information 7 [file peerj-cs-10-2062-s007.zip › Figure 15.png]

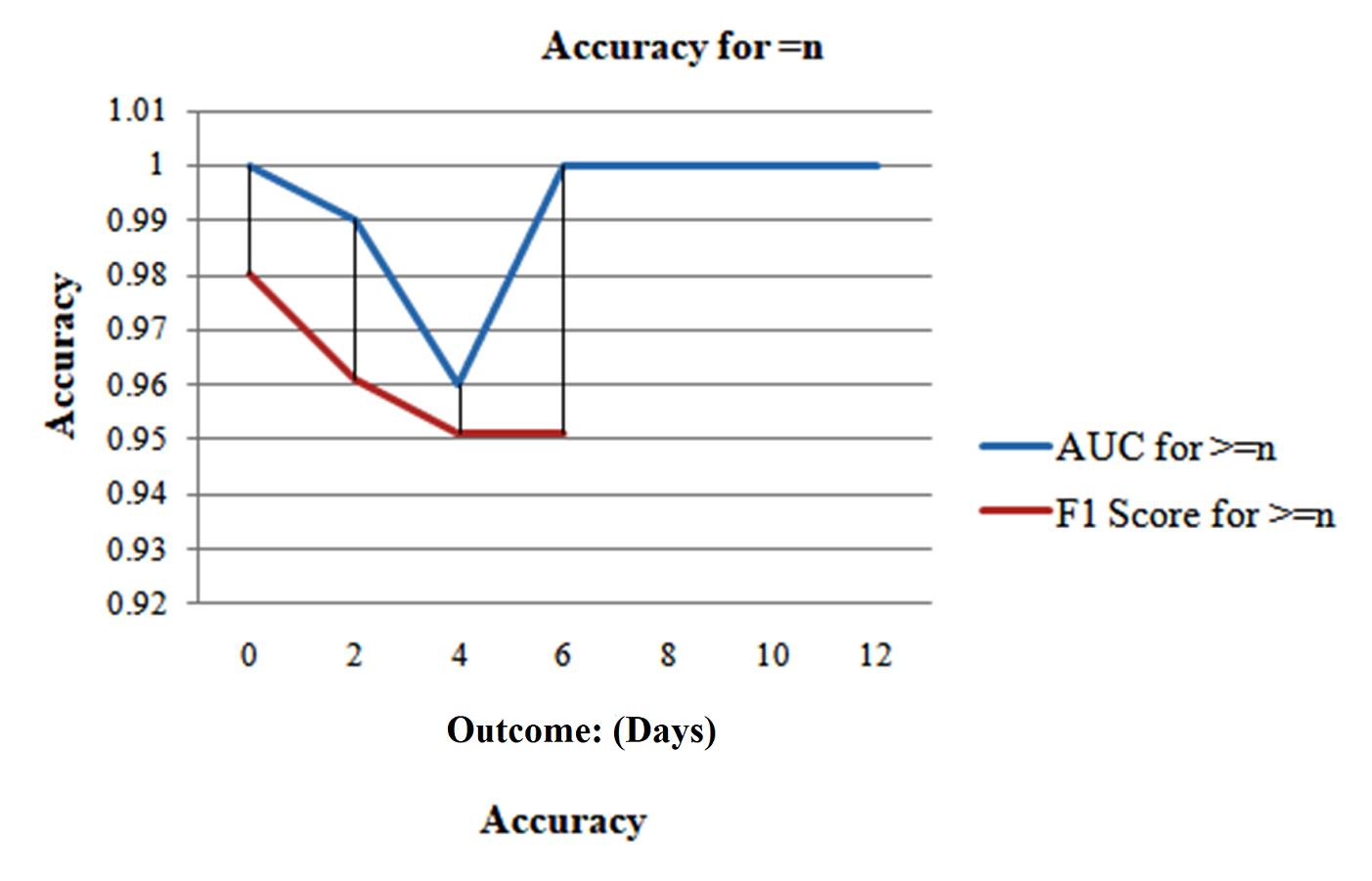

Supplement: Supplemental Information 7 [file peerj-cs-10-2062-s007.zip › Figure 14.png]
